# Nocardiosis: When the Side Effects of Therapy Mimic Symptoms

**DOI:** 10.7759/cureus.25695

**Published:** 2022-06-06

**Authors:** Margarida Silva Cruz, Ligia Rodrigues Santos, Gisela Vasconcelos, Catarina Couto, Tiago Esteves Rodrigues, Rita Veiga Ferraz, Vera Ferraz Moreira, Zélia Lopes, Francisco Cadarso

**Affiliations:** 1 Internal Medicine, Centro Hospitalar Tâmega e Sousa, Penafiel, PRT; 2 Infectious Disease, Centro Hospitalar Tâmega e Sousa, Penafiel, PRT; 3 Pulmonology, Centro Hospitalar Tâmega e Sousa, Penafiel, PRT

**Keywords:** differential diagnosis, therapeutics/adverse effects, trimethoprim-sulfamethoxazole, n. nova/africana, nocardiosis

## Abstract

Nocardiosis is a rare infection caused by gram-positive aerobic actinomycetes, which are common in soil. Inoculation occurs by inhaling agent fragments that cause localized or systemic suppurative lesions. The diagnosis is established based on isolation in cultural examinations. Trimethoprim-sulfamethoxazole (TMP-SMX) is the first-line treatment, and an antimicrobial susceptibility test is useful in severe cases or when there is no clinical response. The duration of treatment is determined by the affected site. However, the treatment cycles are long, and recurrence is common, which has a negative impact on the prognosis.

We describe a case of an immunocompetent male with a recent diagnosis of pulmonary nocardiosis who, after starting therapy, presented with symptoms that could be explained by either disease progression or an adverse pharmacological reaction. Throughout this case, with atypical evolution, the authors review the diagnostic and therapeutic approach to *Nocardia *infection and alert to the importance of the differential diagnosis and available therapeutic options.

## Introduction

*Nocardia (N.)* is an aerobic gram-positive actinomycete that is ubiquitous in soils and was recognized as a pathogen in 1888, but two more years were needed to document the disease in humans [1]. More than 100 species have been described, many of which are associated with human disease, including *N. nova species complexes* [2]. The annual incidence in North America, Europe, and Australia is estimated to be ~0.375 cases per 100,000 population and may be increasing [3]. Inoculation occurs predominantly by inhalation of fragments of this agent, which can cause local (pulmonary nocardiosis) or disseminated (systemic/disseminated nocardiosis) infection. Although nocardiosis typically manifests in immunocompromised patients, up to one-third of cases occur in immunocompetent patients [4].

For the past 50 years, sulfonamides have been the gold standard treatment for nocardiosis. Trimethoprim-sulfamethoxazole (TMP-SMX) is active against most species of *Nocardia*, although resistant *N. nova* is occasionally described [5]. Adverse reactions to this therapy include myelosuppression, hepatotoxicity, and renal failure, among others [6].

## Case presentation

We describe the case of a 55 years-old male bricklayer with a previous medical history of silicosis and treated pulmonary tuberculosis 11 years ago, sensorineural hearing loss, and chronic gastritis. Usual medication included a proton pump inhibitor and an association of inhaled corticosteroids with a long-acting beta-agonist.

**Figure 1:**
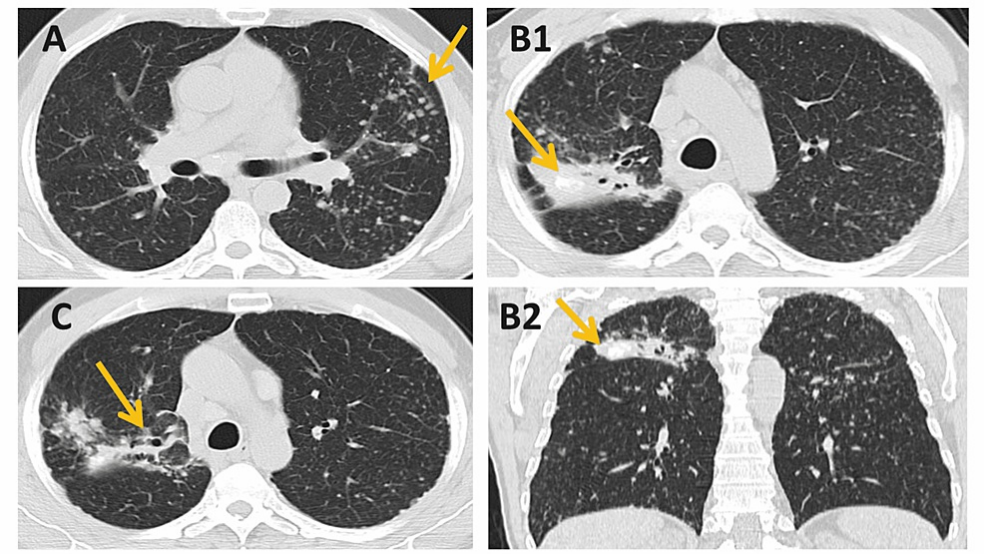
Thoracic computed tomography A - pattern of diffuse centrilobular micronodularity (arrow); B - a consolidative area in the posterior segment of the upper lobe of the right lung, with air bronchogram and calcifications inside (arrow); B1 - axial plane; B2 - coronal plane; C - small bronchiectasis associated with an area of ​​consolidation (arrow)

He presented with hemoptoic cough (one or two episodes/week for four weeks) in a small amount, with no other associated symptoms motivating medical assessment in primary health care. Aminocaproic acid was prescribed, with improvement, and thoracic computed tomography (CT) (Figure 1) revealed “mediastinal adenopathies, a diffuse centrilobular micro-nodularity pattern in all segments, predominantly in the upper lobes, a consolidative area in the posterior segment of the upper lobe of the right lung, with air bronchogram and calcifications inside, suggestive of silicosis/history of tuberculosis. Small bronchiectasis associated with areas of ​​consolidation.”

Due to these findings, he was referred to a consultation where he underwent: bronchoscopy and bronchoalveolar lavage with a collection of samples for bacteriological and mycobacteriological cultures (no significant structural changes were noted). Direct examination, *Mycobacterium tuberculosis* complex deoxyribonucleic acid (DNA) polymerase chain reaction and cytology were negative. Chest CT showed no changes to the previous one.

After three months, a *Nocardia *strain was isolated in the secretions. So, therapy with TMP-SMX 800+160 mg every 12 hours was started. Five days later, due to epigastric pain, the patient decided to reduce the antibiotic to once daily, and afterward, due to generalized tremors and chills without fever assessment, TMP-SMX was discontinued in the absence of medical advice. In the days that followed, he noticed the appearance of maculopapular cutaneous lesions, neither pruritic nor exudative, with initial involvement of the upper limbs and progression to the lower limbs, neck, and face. Due to the worsening of the condition, he turned to the emergency department.

**Figure 2:**
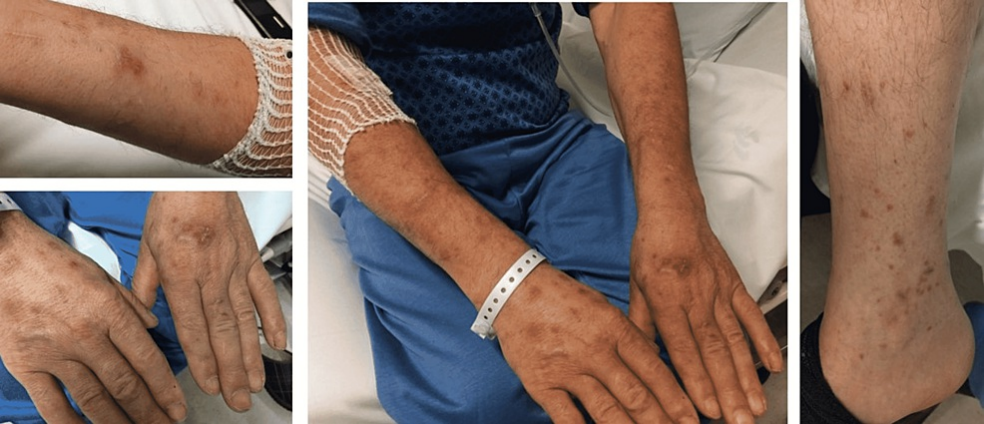
Maculopapular skin lesions

On admission, he denied headaches, focal neurological deficits, weight loss, or other constitutional symptoms. Objectively, he had a fever, was hemodynamically stable, with an oxygen saturation of 93% on room air, and without neurological deficits or meningeal signs. Maculo-papular lesions distributed over the limbs (Figure 2) and neck were noted.

Red eye, with discreet non-purulent exudate on the right, was noted. Pulmonary auscultation with rare snoring and scattered fine crackles. Bloodwork revealed an elevation of systemic inflammatory parameters, elevation of liver cytolysis markers, and coagulopathy (Table 1). There were no other significant changes.

**Table 1:** Baseline laboratory tests ALT, alanine transaminase; AST, aspartate transaminase; CRP, C-reactive protein; INR, international normalized ratio; PT, prothrombin time; WBC, white blood cells

Laboratory findings	Patient values	Reference range
Hemoglobin (g/dL)	12.9	12-15
WBC (counts/µL)	15,910	4,500-11,000
Neutrophils (counts/µL)	14,907	2,000-7,500
Platelet (counts/µL)	265,000	150,000-400,000
CRP (mg/L)	229	<5
ALT (U/L)	226	<31
AST (U/L)	236	<31
PT (seconds)	21.2	11.2
INR	1.88	<1.1

**Figure 3:**
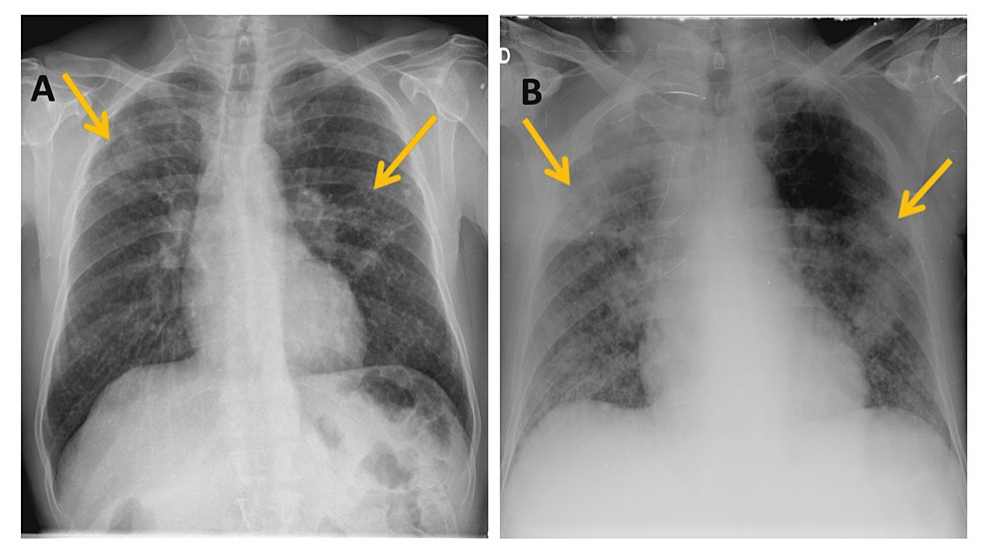
Chest X-rays A - Chest X-ray seven months before hospital admission; B - Chest X-ray in the emergency department, at admission, with worsening diffuse opacities compared to the previous ones (despite increased penetration) (arrows)

Chest X-ray showed worsening diffuse opacities when compared to the previous ones (Figure 3).

Arterial blood gas analysis revealed hypoxemic respiratory failure (Table 2). Brain CT was normal.

**Table 2:** Arterial blood gas on room air HCO3, bicarbonate; Lac, arterial lactate concentration; pCO2, carbon dioxide partial pressure; pO2, oxygen partial pressure; pH, potential of hydrogen; SatO2, arterial oxyhemoglobin saturations

Laboratory findings	Patient values	Reference range
pH	7.48	7.35-7.45
pO2 (mmHg)	60	75-100
pCO2 (mmHg)	40	35-45
HCO3 (mmol/L)	29.8	22-26
SatO2 (%)	92	>95
Lac (mmol/L)	1.7	<2

He was admitted to the internal medicine department and started on amikacin and imipenem (completed 31 days). Human immunodeficiency virus (HIV) serology was negative and peripheral blood immunophenotyping normal, and the remaining results excluded immunosuppression. Ophthalmologic examination, brain magnetic resonance imaging, abdominopelvic CT, and echocardiogram proved to be irrelevant in the clinical context. Skin lesions were not biopsied because they disappeared within the first 24 hours.

After ruling out disseminated disease, it was decided to switch to an oral regimen with trimethoprim/sulfamethoxazole (TMP-SMX), cefixime, and clarithromycin. A few hours after taking TMP-SMX, the patient developed gastrointestinal complaints, shivering, fever, and hypoxemic respiratory failure. Laboratory workup showed an increase in systemic inflammatory parameters, cytocholestasis, and coagulopathy (Table 3).

**Table 3:** Laboratory tests after TMP-SMX ALP, alkaline phosphatase; ALT, alanine transaminase; AST, aspartate transaminase; CRP, C-reactive protein; GGT, gamma-glutamyl transpeptidase; INR, international normalized ratio; LDH, lactate dehydrogenase; PT, prothrombin time; WBC, white blood cells; TMP-SMX: trimethoprim/sulfamethoxazole

Laboratory findings	Patient values	Reference range
Hemoglobin (g/dL)	13.2	12-15
WBC (counts/µL)	14,100	4,500-11,000
Neutrophils (counts/µL)	12,972	2,000-7,500
Platelet (counts/µL)	202,000	150,000-400,000
CRP (mg/L)	237.9	<5
Total bilirubin (mg/dL)	0.8	<1
ALT (U/L)	114	<31
AST (U/L)	74	<31
ALP (U/L)	50	34-104
GGT (U/L)	141	<49
LDH (U/L)	784	266-500
PT (seconds)	25.8	11.2
INR	2.28	<1.1

Supportive therapy was performed, and the previous antibiotic regimen was resumed, with regression of the condition. Identification of the strain revealed *Nocardia Nova/Africana*, and therapy was subsequently switched to amikacin and ceftriaxone according to the antimicrobial susceptibility test. A diagnosis was made of pulmonary nocardiosis caused by *Nocardia Nova/African* associated with a probable adverse reaction to TMP-SMX (Naranjo Adverse Drug Reaction Probability Scale: eight points).

The patient was discharged after 40 days of hospitalization, oriented to Day hospital, for intravenous (IV) administration of amikacin 750 mg/day and ceftriaxone 2 g/day until completing six months of therapy. Response assessment was made by frequent clinical, analytical, and image evaluation (initially biweekly and then monthly).

After six months, the patient presented asymptomatic, with no alterations on examination, blood work with negative systemic parameters and without organ dysfunction, and a slight improvement of radiological abnormalities. Audiometry revealed worsened sensorineural hearing loss with an indication for bilateral hearing aids (which was attributed to amikacin use). Until the present day, the patient remains free from signs of this infection.

## Discussion

We describe a case of *Nocardia *infection in an immunocompetent patient with chronic lung disease, for whom professional activity may have facilitated the inhalation of fragments of this agent through the upper respiratory tract. The association between nocardiosis and other underlying diseases is well-documented, particularly in immunosuppressed states such as HIV, leukemia, post-transplantation, and after prolonged regimens of corticosteroids or cytotoxic therapy [7]. There is no case series in the literature that analyzes the infection by this agent in immunocompetent individuals. Only isolated cases have been reported and mostly affect locations other than the lungs [7]. However, corticosteroid therapy, even a short course, in patients with chronic obstructive pulmonary disease seems to be another predisposing risk factor for this infection [8].

The characteristic histological findings of this disease are an abscess with extensive neutrophil infiltration and prominent necrosis due to *Nocardia*’s ability to survive within phagocytes [3]. Diagnosis is established after identification of the agent on microscopic and cultural evaluation (fastidious growth, two to four weeks). Biopsy or culture of exudate from skin lesions or brain imaging may also be considered to exclude dissemination [3,7].

In the respiratory system, Nocardiosis can manifest itself more frequently as pneumonia with cough, thick purulent secretions, fever, anorexia, weight loss, malaise (common); dyspnea, hemoptysis, and pleuritic pain (less common). Chest radiography shows single or multiple infiltrates that tend to cavitate [7]. In one-third of cases, it complicates with empyema and more than 50% have extrapulmonary involvement [4]. Less common respiratory manifestations are laryngitis, tracheitis, bronchiolitis, and sinusitis [3]. Endophthalmitis, keratitis (uncommon), and lacrimal gland involvement may occur [3]. Cellulitis, lymphocutaneous disease, and actinomycetoma can occur after transcutaneous inoculation [1]. The extrapulmonary disease can involve the brain (most common), skin, kidneys, bones, eyes, muscles, and meninges. Thus, infections by other bacteria, mycobacteria, fungi (aspergillosis, mucormycosis, histoplasmosis, cryptococcosis), neoplasms, or, in the case of central nervous system involvement, cerebral infarction, constitute differential diagnoses for nocardiosis [4].

In the beginning, clinical findings presented by this patient were interpreted as systemic/disseminated nocardiosis, so it was decided to start combined therapy according to recommendations. After ruling out disseminated disease and clinical improvement, it was decided to change therapy to an oral regimen, and TMP-SMX was reintroduced. At that time, the patient experienced clinical worsening compatible with TMP-SMX adverse reaction.

This drug is the treatment of choice in most cases, however, there is a lack of prospective randomized trials to validate it. Variable patterns of susceptibility of *Nocardia *to antimicrobials have been reported in vitro, so treatment should be individualized [4]. Adverse reactions to TMP-SMX include myelosuppression, hepatotoxicity, renal failure, and symptoms such as epigastric pain, skin rash, red eye, fever, hypotension, dyspnea with hypoxia, among others, are also described, although their frequency is rare [4,6].

Combination therapy with TMP-SMX, amikacin, and ceftriaxone or imipenem is recommended in severe disease [7]. antimicrobial susceptibility test is advisable in cases of severe/widespread infection, refractoriness to therapy, or intolerance to sulfonamides [7]. The surgical approach, when necessary, is similar to other bacterial diseases [3]. The duration of treatment depends on the site of the infection (Table 4), and recurrence is common, so follow-up is recommended for at least six months after the end of antibiotic therapy [3-4].

**Table 4:** Duration of antibiotic treatment in Nocardiosis according to the location of the disease (a): HIV + CD4 < 200/uL or chronic granulomatous disease: treatment continued indefinitely; (b): Total removal of pyogenic material: therapy can be shortened six months [3]

Disease site	Duration
Cellulitis, Lymphocutaneous syndrome	2 months
Osteomielite, artrite, laringite e rinossunusite	4 months
Keratitis	Topic: until cure; Systemic: 2-4 months after healing
Actinomycetoma	6-12 months after cure
Pulmonary or systemic	Immunocompetent: 6-12 months; Immunocompromised: 12 months (a); Central nervous system: 12 months (b)

When properly treated, the mortality rate of disseminated and pulmonary, excluding the central nervous system, is less than 5% [3].

In the presented case, and after the antimicrobial susceptibility test result, it was decided to maintain combined therapy, and it wasn't possible to change to an oral regimen. The clinical evolution was favorable, and until this date, there hasn't been any documented recurrence.

## Conclusions

We described an unusual case of pulmonary nocardiosis caused by *Nocardia nova/Africana,* in an immunocompetent individual, associated with an adverse reaction, also rare, to the TMP-SMX therapy. Throughout it, we not only present a review of the diagnostic and therapeutic approach to* Nocardia* infection, but we also alert to possible adverse effects related to the treatment. Facing a clinical worsening, we should go back to the beginning and rethink the diagnosis. Possible differential diagnoses must be questioned without never forgetting the adverse effects of drugs or patient allergies as a possible cause.
